# Safety and Survival Rate of a Biomimetic Osteochondral Scaffold for the Treatment of Lesions of the Knee Articular Surface

**DOI:** 10.1177/23259671261418010

**Published:** 2026-03-24

**Authors:** Luca Andriolo, Luca De Marziani, Alessandro Di Martino, Angelo Boffa, Lorenzo Zanasi, Giacomo Roveda, Roberta Miale, Stefano Zaffagnini

**Affiliations:** * Clinica Ortopedica e Traumatologica 2, IRCCS Istituto Ortopedico Rizzoli, Bologna, Italy; ‡ Applied and Translational Research (ATR) Center and Clinica Ortopedica e Traumatologica 2, IRCCS Istituto Ortopedico Rizzoli, Bologna, Italy; § Department of Clinical Affairs, Finceramica Faenza spa, Faenza, Italy; Investigation performed at Finceramica Faenza spa, Faenza, Italy

**Keywords:** safety, osteochondral scaffold, adverse event, failure rate

## Abstract

**Background:**

A cell-free biomimetic osteochondral scaffold has been developed to treat lesions of articular knee surface, with good outcomes documented up to long-term follow-up. However, systematic tracking of failures and adverse events (AEs) that might affect the outcome is still missing.

**Purpose:**

To analyze the incidence of AEs and failures at long-term follow-up in a large series of patients after knee cell-free scaffold implantation.

**Study Design:**

Case series; Level of evidence, 4.

**Methods:**

The database with the regulatory-required safety data collected by the manufacturer was utilized. A total of 704 patients with a mean follow-up of 44.8 months (range, 6-180) were included. Data from implantation centers were analyzed focusing on AEs and failures. AEs were divided by the surgeons into surgery related, treatment related, or not related to surgery or treatment, and into serious or mild AEs. Additionally, factors associated with AEs and failures were examined.

**Results:**

A total of 177 postoperative AEs were reported in 170 patients (24.1%). Among these, surgeons considered 104 AEs surgery related and 73 treatment related. Moreover, for 45 patients (6.4%), 52 AEs not related to surgery or treatment were reported, which were excluded from the analysis. Mild AEs were reported in 93 patients (13.2%), including minor AEs in the early or postoperative period (such as persistent postoperative symptoms, such as fever, pain, knee swelling, or difficulty of movement) in 82 patients (11.6%). Serious AEs were reported in 66 patients (9.4%), including early or persistent postoperative symptoms in 54 patients (7.7%), joint inflammation in 6 (0.9%), arthrofibrosis in 3 (0.4%), and 0.4% in others. Finally, this scaffold presented a survival rate of 93.1%, according to life table method, (31 surgical failures) at up to 180 months’ observation. Demographic and clinical patient characteristics did not significantly influence the risk of postoperative AEs or failures.

**Conclusion:**

The implantation of this scaffold for the treatment of knee articular surface lesions presents a high survival rate of 93.1% at long-term follow-up. Mild AEs (13.2%) and serious AEs (9.4%) reported by the treating surgeons in the database consisted mainly of pain and postoperative symptoms. These results can help surgeons to improve the clinical decision-making process and the patient counseling regarding expectations related to the implantation of an osteochondral scaffold to treat articular knee surface lesions.

Lesions of the knee articular surface represent a frequent pathology that affects >60% of patients undergoing knee arthroscopy.^15,39^ If not adequately treated, these lesions can lead to chronic symptoms such as swelling and pain and to the development of early osteoarthritis.^16,26,33^ Over the years, several treatments have been developed to address knee cartilage lesions, including bone marrow stimulation techniques, osteochondral autograft or allograft transplantation, and autologous chondrocyte–based procedures.^4,21,22^ However, these treatments had several disadvantages, including inadequate repair tissue quality, donor-site morbidity, limited availability, inability to treat the entire osteochondral unit, 2-stage procedures, or significant costs associated with not always satisfactory clinical outcomes.^9,14,28,36^ Cell-free osteochondral scaffolds have been introduced to overcome these limitations, providing a temporary biodegradable 3-dimensional structure to promote tissue growth and, ultimately, regeneration of the osteochondral unit.^3,7,8,11,13^

Osteochondral scaffolds have shown good clinical results up to long-term follow-up with a low failure rate, even in patients with complex injuries affected by large defects, difficult locations, and early osteoarthritis.^10,12,35^ However, along with the good clinical results, postoperative adverse events (AEs) following cell-free osteochondral scaffold implantation have been reported in more than 30% of patients.^10,31^ Systematically tracking AEs and failures is mandatory for medical companies and helps surgeons to better understand specific risk factors, allowing them to develop preventive measures and safety protocols to reduce complications.^6^ To understand the significance of AEs and failures, large series with long-term follow-ups are needed. Studies reporting postoperative AEs after cell-free osteochondral scaffold implantation are generally small series and long-term follow-ups are rare. Moreover, the incidence of minor self-resolving complications, which are burdensome for the patients, affecting their quality of life (especially in the early postoperative period) is frequently not accurately evaluated. Finally, the current literature does not allow the identification of patient-specific risk factors, hindering the possibility of tailoring the treatment to their risk profile for osteochondral scaffold implantation.

The aim of this study was to analyze the incidence of AEs and failures at long-term follow-up and to investigate their association with patient and lesion characteristics in a large series of patients after knee cell-free osteochondral scaffold implantation.

## Methods

The database with the required regulatory safety data collected by the scaffold manufacturer (Finceramica Faenza spa) was used for this study. The current Medical Device Regulation (2017/745; EU) considers the clinical follow-up of medical devices on the market as a process aimed at continuously updating the clinical evaluation of a device. For this reason, proactive vigilance/surveillance activities are periodically carried out, with the aim of ensuring the long-term follow-up safety of the implanted devices by raising surgeons’ awareness of the importance of accurately reporting to the company any data regarding implant safety and survival at each follow-up visit. The total population consisted of 1397 patients with 1486 lesions treated with a cell-free biomimetic osteochondral scaffold (MaioRegen; Finceramica Faenza spa) for lesions of the articular surface. Detailed patient characteristics are shown in Table 1.

**Table 1:** Detailed Characteristics of Treated and Included Patients*^a^*

	Treated Patients	Data Available, n/N (%)*^b^*	Included Patients	Data Available, n/N (%)*^b^*
Sex, M/F	945/406	1351/1397 (96.7)	487/194	681/704 (96.7)
Age, y	35.9 ± 12.6	1350/1397 (96.6)	34.1 ± 12.1	675/704 (95.9)
Lesion size, cm^2^	3.5 ± 2.0	1010/1397 (72.3)	3.7 ± 2.0	483/704 (68.6)
Concomitant knee surgery, yes/no	369/1028	1397/1397 (100)	292/412	704/704 (100)
Lesion location	CFM: 704 CFL: 225 Patella: 215 Trochlea: 197 Tibial plateau: 49	1390/1486 (93.5)	CFM: 320 CFL: 118 Patella: 138 Trochlea: 121 Tibial plateau: 23	720/764 (94.2)
Number of lesions	Single: 1313 Multiple: 84	1397/1397 (100)	Single: 648 Multiple: 56	704/704 (100)
Etiology	Traumatic: 312 Degenerative: 478 OCD: 335 Osteonecrosis: 12 Other: 61	1198/1486 (80.6)	Traumatic: 145 Degenerative: 278 OCD: 158 Osteonecrosis: 10 Other: 43	634/764 (83.0)
Type of scaffold	Prime: 1156 Slim: 173 Chondro+: 68	1397/1397 (100)	Prime: 567 Slim: 86 Chondro+: 51	698/704 (99.1)

a Values are expressed as n, mean ± SD, or n/N (%).CFL, lateral femoral condyle; CFM, medial femoral condyle; F; female; M, male; OCD, osteochondritis dissecans. Only data available in the registry have been reported on the table; consequently, not always the total number of patients is included, and the exact number of patients analyzed is reported for both treated and included patients.

b Values are presented as n/N, where n denotes the number of available observations for the specific variable within each category and N denotes the corresponding reference population. For treated cases, N = 1397 for patient-based variables (total number of treated patients) and N = 1486 for lesion-based variables (total number of treated lesions). For the follow-up analysis, N = 704 for patient-based variables (patients with available follow-up) and N = 764 for lesion-based variables (lesions with available follow-up).

Inclusion criteria were patients with clinical symptoms related to chondral or osteochondral lesions involving the femoral condyles, trochlea, patella, or tibial plateau that underwent scaffold implantation between January 2007 and January 2024 and with ≥6 months of follow-up. The data from the centers where the scaffold implantation was performed were analyzed focusing on AEs^37^ and failures defined as procedures involving the removal of the implant. AEs were divided into (1) related to surgery, (2) related to treatment, or (3) not related to surgery or treatment and were also divided into serious or mild AEs. Additionally, potential factors associated with AEs and failures were examined.

**Figure 1.**
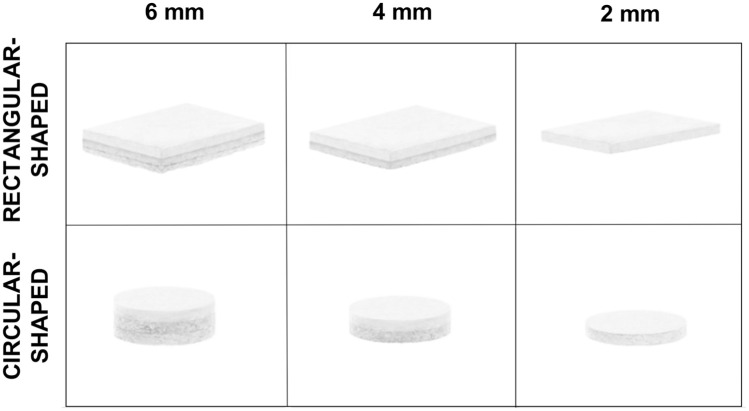
Different available scaffolds: “Prime”, “Slim” and “Chondro+” in the rectangular- and the circular-shaped formulations.

The MaioRegen osteochondral biomimetic scaffold is a 3-layer, 3-dimensional structure composed of type 1 equine collagen and hydroxyapatite, which mimics the osteochondral anatomy. Three formulations are available on the market and have been studied within the database: “Prime,”“Slim,” and “Chondro+.” MaioRegen “Prime,” the first developed implant, is a 3-layer scaffold with a 6-mm depth (Figure 1). The cartilaginous layer, composed of equine type 1 collagen, presents a smooth surface to facilitate joint surface gliding. The middle layer, designed to simulate the cartilage tide mark, consists of 60% type 1 collagen and 40% hydroxyapatite (HA). The bottom layer replicates the subchondral bone, comprising a mineralized blend of 30% type 1 collagen and 70% HA. MaioRegen "Slim" is a 4-mm deep, double-layer scaffold specifically designed for the treatment of chondral and less deep osteochondral lesions. The cartilaginous layer is 100% composed of equine type 1 collagen, while the bottom layer consists of 60% type 1 collagen and 40% HA. MaioRegen "Chondro+" is a 2-mm deep scaffold designed for treating chondral lesions with minimal or no involvement of the subchondral bone. It is composed of a 2-mm cartilaginous layer made of equine type 1 collagen, with a very thin coating of 60% type 1 collagen and 40% HA on the part interfacing with the subchondral bone.

All patients underwent cell-free biomimetic scaffold implantation using a previously described surgical technique.^1^ After applying a tourniquet to the proximal thigh, the surgical approach was determined by the lesion's location, with the incision made medially or laterally to the patella through the skin, subcutaneous layers, and joint capsule. Once the chondral or osteochondral lesion was exposed, the site was carefully prepared by removing the damaged osteochondral tissue to create a defect of precise depth suitable for scaffold placement. The procedure can be performed with osteotomes or with a dedicated instrumentation.^1^ The scaffold size was selected to match the prepared area accurately. Following this, the scaffold was implanted using a press-fit technique. To enhance stability, fibrin glue could be applied according to surgeon preference.^1^

### Statistical Analysis

Variables collected were subjected to descriptive analyses, including arithmetic mean, median and standard deviation, or range for quantitative variables and reporting count and percentages for noncontinuous variables. Graphical methods were used to explore deviations from normal distribution of the continuous variables. The life table method was used to estimate probability of surviving without surgical failure. Eventual influence of some risk factors on cumulative incidence of AE, of serious AE, or of explantation was explored via Fisher exact test when evaluating the individual risk factor alone, and via logistic regression when evaluating risk factors with a multifactorial approach. Statistical analyses were performed using SAS (SAS Statistical Software; SAS Institute).

## Results

According to the inclusion criteria, 704 patients with ≥6-month follow-up were included in the analysis, representing 50.4% of the total population. Detailed characteristics of the included patients and comparison with the entire population are shown in Table 1. Patients had a mean follow-up of 44.8 months (range, 6-180; median, 24) after surgery.

A total of 177 postoperative AEs were reported in 170 patients (24.1%). Among these, surgeons considered 104 AEs related to surgery and 73 related to treatment. Moreover, for 45 patients (6.4%), 52 AEs not related to surgery or treatment were reported, which were not included in the following analysis. Mild AEs, as defined by the treating surgeons, were reported in 93 patients (13.2%) including minor AEs in the early or postoperative period (such as persistent postoperative symptoms, such as fever, pain, knee swelling, or difficulty of movement) in 82 patients (11.6%), joint inflammation in 4 patients (0.6%), joint crepitus in 3 patients (0.4%), and 1 loose body (0.1%). Serious AEs, as defined by the treating surgeons, were reported in 66 patients (9.4%) including early or persistent postoperative symptoms (eg, fever, pain, knee swelling, or difficulty of movement) in 54 patients (7.7%), joint inflammation in 6 patients (0.9%), arthrofibrosis in 3 (0.4%), joint crepitus in 1 (0.1%), 1 loose body (0.1%), 1 patellar tendinitis (0.1%), and 1 early resorption (0.1%). In 4 cases, the surgeons did not declare the type of AE and in 12 cases the severity of the event was reported as an unknown AE.

The occurrence of AEs decreased over time following the surgical procedure (data about timing of AEs occurring in 134 patients, 134/170 (78.8%) of patients with AEs). Specifically, 81.3% of adverse events were reported within the first 6 months, 7.5% occurred between 7 and 12 months, 6.7% between 13 and 24 months, and 4.5% after 24 months of follow-up.

**Figure 2.**
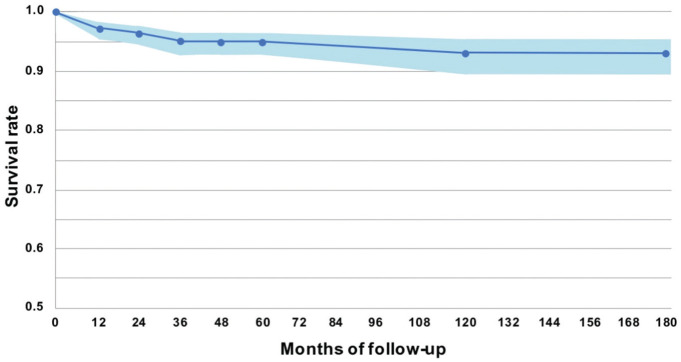
Survival rate for patients treated with osteochondral scaffolds (blue line) ± 95% CI (light blue line).

A total of 31 patients during 180 months of observation were considered surgical failures according to the definition. In detail, 22 patients failed before 24 months of follow-up, 6 patients failed between 24 and 60 months of follow-up, and 3 patients between 60 and 180 months of follow-up. Figure 2 shows the probability estimates according to life table method, and Table 2 shows the probability of failure at the defined intervals.

**Table 2:** Probability of Explantation Across the Different Time Intervals Analyzed*^a^*

Patients Evaluated	Follow-up Range (months)	SD Estimate	SD Lower CL	SD Upper CL
704	00 to <1	1.00	N/A	N/A
704	≥1 to <6	1.00	N/A	N/A
704	≥6 to <12	1.00	N/A	N/A
577	≥12 to <24	0.97	0.96	0.98
470	≥24 to <36	0.96	0.95	0.98
321	≥36 to <48	0.95	0.93	0.97
245	≥48 to <60	0.95	0.93	0.97
207	≥60 to <120	0.95	0.93	0.97
90	≥120 to <180	0.93	0.90	0.95
23	≥180	0.93	0.90	0.95

a CL, confidence limit; N/A, not applicable; SDF, Survival Distribution Function.

**Figure 3.**
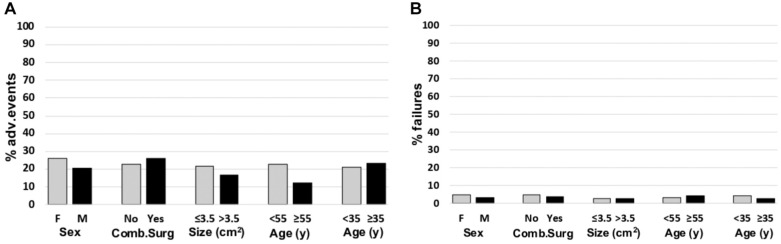
Percentage of patients with (A) adverse events (AEs) and (B) failure rates for the different analyzed characteristics. Comb.Surg, combined surgery; F, female; M, male.

A further analysis was performed to evaluate the influence of patient characteristics on the risk of serious AEs and implant failure (Figure 3).

All demographic and clinical patient characteristics, such as sex, age, combined knee surgeries, etiology, lesion site, and lesion size did not influence the risk of postoperative AEs. Furthermore, sex, age, combined knee surgeries, etiology, lesion site, and lesion size did not statistically influence the risk of implant failure.

## Discussion

The main finding of the study is that biomimetic osteochondral scaffolds implanted for the treatment of knee chondral and osteochondral lesions resulted in a high survival rate of 93.1% at up to 180 months follow-up, and an AE rate of 24.1%, with 9.4% of serious AEs related to surgery or treatment. Demographic and clinical patients’ characteristics did not show to influence the risk of postoperative AEs or failures.

The implant survival rate demonstrated by the current study is >90% and confirms previously reported studies, which mainly included smaller series evaluated at shorter follow-ups. A recent prospective study conducted on >200 patients treated with this osteochondral scaffold demonstrated that the surgical failure rate is approximately 2% at 2 years of follow-up.^10^ Moreover, another prospective study on 24 patients evaluating the survival of this implant at 10 years of follow-up found failure rates similar to those of the current study (4%).^12^ The current study adds important data to the literature, confirming in a large series and at long-term follow-up the high postoperative survival rate of this osteochondral scaffold. Postoperative survival of any surgical treatment is a critical aspect that surgeons must consider when counseling a patient, as reduced treatment survival necessitates additional surgical procedures, increases costs and overall hospital stay, and, most importantly, worsens clinical outcomes while raising the risk of postoperative complications.^2,30^

The risk of AEs is another key aspect that should be considered and discussed when counseling patients for surgery. The most frequent serious AE reported by the treating surgeons after the implantation of this cell-free scaffold was the presence of minor or persistent postoperative symptoms (fever, pain, knee swelling, or difficulty of movement), observed in 7.7% of the treated patients, as defined by the surgeons. The incidence of chronic pain following scaffold implantation is comparable with other common surgical knee procedures. For example, previous studies showed that chronic pain can affect >20% of patients undergoing anterior cruciate ligament reconstruction.^18,29,38^ Moreover, chronic pain is frequent even after arthroscopic knee procedures, with incidence ranging from 6% to 25% at 1 and 2 years of follow-up, respectively.^20,34,40^ Another serious AE reported was arthrofibrosis, which occurred in 0.4% of the cases. A possible explanation for the development of postoperative arthrofibrosis could be joint bleeding resulting from subchondral bone exposure during the procedure, leading to hemarthrosis and subsequent fibrotic tissue formation. To mitigate this risk, drainage of hematoma when present and early postoperative mobilization could be effective preventive measures. It is also important to consider that, although arthroscopic implantation techniques for this cell-free scaffold have been recently described in the literature,^17^ the standard technique involves an arthrotomy, which could play a role in the onset of this type of complication. Nevertheless, the risk of postoperative arthrofibrosis in this context remains lower than that associated with other common open knee surgical procedures.^24,27,32^ It is also important to note that mild adverse events are relatively common following implantation of this scaffold, occurring in 13.2% of patients. However, previous studies demonstrated that, although these events may delay recovery in the early postoperative period, they do not affect patient outcomes at the 2-year follow-up.^10^

Another important finding of the current study is that lesion size did not significantly influence the risk of postoperative AEs. This is particularly important, as previous studies showed that this cell-free scaffold can achieve good clinical outcomes even in patients with large chondral and osteochondral lesions.^10,35^ Although the use of chondral and osteochondral scaffolds is usually recommended for medium-small lesions <4 cm^2^,^5,19^ a recent consensus highlighted their appropriateness also for the treatment of large articular surface lesions.^13^ It is worth noticing that larger lesions are commonly associated with other comorbidities, including meniscal lesion, ligament instability, and malalignment, needing to be addressed surgically.

In this perspective, this cell-free scaffold could be a reliable option for the treatment of large chondral and osteochondral lesions when concomitant procedures are needed, as demonstrated by the current study showing no influence of concomitant procedure on the risk of postoperative AEs and failures. Several studies demonstrated that the clinical outcome of cartilage repair procedures improves if patient- and joint-specific factors are addressed in the treatment algorithm of cartilage defects.^16,23,41^ For these reasons, surgical procedures associated with cartilage treatments are performed in approximately half of cases.^41^ Previous studies described the use of this cell-free scaffold associated with other surgical procedures to treat early osteoarthritis and reported good results, especially in patients <40 years of age.^25,35^ A recent article by Solaro et al^35^ on 39 patients affected by early osteoarthritis and treated with osteochondral scaffold and concomitant procedures such as osteotomy, meniscal transplant, and meniscal replacement demonstrated 5% of surgical failures and a significant clinical improvement compared with baseline at a minimum 10-year follow-up. Hence, there is a possibility of utilizing these types of treatments even in complex cases in an attempt to postpone more invasive procedures, especially in younger patients where the chances of recovery are greater and the risk of postoperative AEs is lower.

### Limitations

The current study presents some limitations. First of all, only AEs and failures could be evaluated because of the nature of the study while patient-reported outcome measures or imaging findings were not available. Furthermore, it is important to underline the heterogeneity of the clinical characteristics of the patients evaluated, particularly in terms of etiology, lesion size, site, and age. Another limitation of the study is the relatively low proportion of patients evaluated at follow-up and, in a few cases, the absence of detailed follow-up data, particularly regarding the severity and type of AEs reported by the surgeons.

## Conclusion

The implantation of this biomimetic osteochondral scaffold for the treatment of knee articular surface lesions presents a high survival rate of 93.1% up to long-term follow-up. Mild AEs (13.2%) and serious AEs (9.4%) reported by the treating surgeons in the database consisted mainly of pain and postoperative symptoms. Demographic and clinical patients’ characteristics did not influence the risk of postoperative AEs or failures. These results can help surgeons to improve the clinical decision-making process and the patient counseling regarding expectations related to the implantation of an osteochondral scaffold to treat chondral and osteochondral knee lesions.
